# Genistein‐3′‐sodium sulphonate protects against lipopolysaccharide‐induced lung vascular endothelial cell apoptosis and acute lung injury via BCL‐2 signalling

**DOI:** 10.1111/jcmm.14815

**Published:** 2019-11-22

**Authors:** Lei Yi, Mengling Chang, Quanming Zhao, Zengding Zhou, Xiaoqin Huang, Feng Guo, Jingning Huan

**Affiliations:** ^1^ Department of Burn and Plastic Surgery School of Medicine Ruijin Hospital Shanghai Jiao Tong University Shanghai China; ^2^ Department of Orthopedic Surgery The Second Affiliated Hospital of Nantong University Nantong Jiangsu Province China; ^3^ Department of Plastic Surgery Shanghai Jiaotong University Affiliated Sixth People's Hospital Shanghai China

**Keywords:** acute lung injury, apoptosis, endothelial cells, genistein‐3′‐sodium sulphonate, sepsis

## Abstract

Under septic conditions, Lipopolysaccharide (LPS)‐induced apoptosis of lung vascular endothelial cells (ECs) triggers and aggravates acute lung injury (ALI), which so far has no effective therapeutic options. Genistein‐3′‐sodium sulphonate (GSS) is a derivative of native soy isoflavone, which has neuro‐protective effects through its anti‐apoptotic property. However, whether GSS protects against sepsis‐induced lung vascular endothelial cell apoptosis and ALI has not been determined. In this study, we found that LPS‐induced Myd88/NF‐κB/BCL‐2 signalling pathway activation and subsequent EC apoptosis were effectively down‐regulated by GSS in vitro. Furthermore, GSS not only reversed the sepsis‐induced BCL‐2 changes in expression in mouse lungs but also blocked sepsis‐associated lung vascular barrier disruption and ALI in vivo. Taken together, our results demonstrated that GSS might be a promising candidate for sepsis‐induced ALI via its regulating effects on Myd88/NF‐κB/BCL‐2 signalling in lung ECs.

## 1 INTRODUCTION

Sepsis is a severe life‐threatening syndrome of multiple organ dysfunction induced by dysregulated host immunoreaction to gram‐negative bacterial infection.^1^ Mortality in sepsis is mainly due to multiple organ dysfunction syndrome, of which acute lung injury (ALI) is a common and critical component.^2^ Lipopolysaccharide (LPS), as a major component of the outer membrane of gram‐negative bacteria, most frequently induces dysfunction of the endothelial cells (ECs), which line the intimal surface of the vasculature.^3^ LPS‐induced lung vascular EC apoptosis is the predominant mechanism for the development of ALI in sepsis.^4^, ^5^, ^6^ However, there are no specific pharmacological therapies for LPS‐induced EC apoptosis and ALI in sepsis.

BCL‐2 family proteins play an important role in cellular apoptosis process by regulating mitochondrial membrane permeability.^7^ NF‐κB regulates BCL‐2 family proteins in immune responses,^8^ and inhibition of NF‐κB activity has been shown to protect against LPS‐induced EC apoptosis.^9^ Interestingly, our previous studies revealed that LPS‐induced activation of NF‐κB in ECs was mediated in a Myd88‐dependent manner and a Myd88‐independent manner (GEF‐H1 signalling pathway).^10^, ^11^ Both Myd88 and GEF‐H1 signalling also were implicated in the regulation of apoptosis and proliferation in many cells.^12^, ^13^ Hence, LPS‐mediated activation of Myd88/NF‐κB pathway or GEF‐H1/NF‐κB pathway might be involved in EC apoptosis by regulating the expression of BCL‐2 family proteins. Blocking the activation of these signalling pathways might inhibit the progression of EC apoptosis and ALI in sepsis.

Genistein is a native isoflavone extracted from food sources such as tofu and beans,^14^ which has significant anti‐inflammatory and antioxidant pharmacological properties.^15^, ^16^ Importantly, a recent study observed that genistein‐3′‐sodium sulphonate (GSS), a new derivative of genistein, has the anti‐apoptotic activity in focal cerebral ischaemia‐induced neuronal injury by inhibiting BCL‐2 family‐associated activation of the apoptotic pathway.^17^ These findings suggested that GSS might be involved in the regulation of LPS‐induced EC apoptosis processes.

Based on the above data, we aimed to determine whether GSS protects against LPS‐induced EC apoptosis and to explore the underlying molecular mechanisms. In the present study, we showed that GSS effectively inhibited LPS‐induced Myd88/NF‐κB/BCL‐2/caspase‐3 signalling activation in ECs, which contributed to protection against LPS‐induced lung vascular EC apoptosis. Furthermore, we found that GSS not only significantly reversed sepsis‐induced BCL‐2 decreases in mouse lung tissue but also inhibited lung vascular barrier disruption and ALI in sepsis. Our findings provide new insight into the protective role of GSS in LPS‐induced lung vascular EC apoptosis, which may be a novel therapeutic method for lung vascular permeability and ALI in sepsis.

## 2 MATERIALS AND METHODS

### 2.1 Cell culture and main reagents

Primary mouse pulmonary micro‐vascular endothelial cells (MPMECs) were obtained Angio‐Proteomie and maintained in Angio‐Proteomie Endothelial Cell Medium in a humidified 37°C, 5% CO_2_ incubator. GSS was obtained from Shanghai Tian Xi Chemical Co., Ltd., and BAY11‐7082 was purchased from Beyotime of China. The LPS was obtained from Sigma‐Aldrich. Anti‐GEF‐H1 rabbit monoclonal antibody (mAb), anti‐Myd88 rabbit mAb, anti‐NF‐κB p65 rabbit mAb, anti‐rabbit phospho‐NF‐κB‐p65 rabbit antibody, anti‐BCL‐2 rabbit mAb and anti‐PARP rabbit mAb were obtained from Cell Signaling Technology. Anti‐CD31 antibody and anti‐cleaved‐PARP antibody were purchased from Santa Cruz Biotechnology. The Alexa Fluor 555‐conjugated goat antimouse secondary antibody and Alexa Fluor 594‐conjugated goat anti‐rabbit secondary antibody and the ProLong Gold Antifade Mountant with DAPI were obtained from Invitrogen Life Sciences.

### 2.2 Cell viability assay

MPMEC viability was checked by a Cell Counting Kit‐8 (CCK‐8; CK04, DOJINDO) according to the manufacturer's protocol. MPMEC suspensions were added to 96‐well plates at a density of 1 × 10^4^ cells/mL and cultured for 24‐48 hours. The MPMECs were then exposed to various concentrations of GSS for different times. Next, a total of 10 μL CCK‐8 solution was added to each well, and the MPMECs were incubated for an additional 3 hours. Finally, the absorbance at 450 nm was measured using a microplate reader (Thermo Fisher Scientific, Inc).

### 2.3 Markers of apoptosis (ECs and lung sections)

MPMECs were incubated separately with medium, LPS, GSS or both LPS and GSS for the indicated times. A total of 5 × 10^5^ cells/mL were harvested and suspended in 500 μL binding buffer. Subsequently, cells apoptosis was quantified by staining with 5 μL of Annexin V‐fluorescein isothiocyanate and 5 μL of propidium iodide (PI) for 15 minutes in the dark, and the results were analysed by FlowJo software (LLC). Apoptosis of lung sections was measured by terminal deoxynucleotidyl transferase‐mediated dUDP nick‐end labelling (TUNEL) staining (Roche). TUNEL‐positive cells were imaged under a fluorescence microscope. Cells showing green fluorescence were considered apoptotic cells.

### 2.4 Extraction of nuclear and cytosolic fractions

Nuclear and cytoplasmic protein extraction was performed according to the manufacturer's instructions for a nuclear and cytoplasmic protein extraction kit (Beyotime). After stimulation, ECs were washed and collected by centrifugation, and then, MPMEC pellets were re‐suspended in extraction buffer A and incubated for 15 minutes on ice before extraction buffer B was added. After centrifugation, supernatants containing cytoplasmic proteins were removed and stored at −80°C. The cell pellets were re‐suspended in nuclear extraction buffer, and the nuclear proteins were extracted by shaking the samples. Afterwards, samples were centrifuged and the supernatants were removed and stored. Finally, the nuclear and cytoplasmic proteins were checked using Western blot analysis (histone‐H3 was used as a loading control for nuclear proteins).

### 2.5 Western blot assay

Proteins were obtained from MPMECs or lung tissue extracts, and then, target proteins were probed with specific antibodies. After the proteins were separated by 6%‐12% sodium dodecyl sulphate polyacrylamide gel electrophoresis (SDS‐PAGE) and electro‐transferred to PVDF membranes, the membranes were incubated with antibodies to the target proteins (GEF‐H1, Myd88, BCL‐2, PARP, NF‐κB p65 and P‐NF‐κB p65, diluted to 1:1000; the anti‐cleaved‐PARP antibody dilution was 1:500) overnight at 4°C, and then, the bands were scanned using a gel image processing system (Image Pro Plus v7.0; Media Cybernetics, Inc). GAPDH was used as a loading control for proteins.

### 2.6 Transfection with Myd88 siRNA in vitro

The Myd88 siRNA was designed and synthesized by Gene Pharma Technologies. Myd88 siRNA was transfected into MPMECs using Lipofectamine 3000, and the ability of RNA interference molecules to knockdown target protein was analysed by Western blot analysis. The siRNA sequence targeting Myd88 was as follows: 5′‐CCG GAU GGU GGU GGU UGU CUC UGA U‐3′. The negative control sequence was as follows: 5′‐UUC UCC GAA CGU GUC ACG UTT‐3′.

### 2.7 Confocal immunofluorescence analysis

To detect active NF‐κB, MPMECs were fixed in 4% paraformaldehyde for 20 minutes and then permeabilized with 0.1% Triton X‐100 in 1% bovine serum albumin for 15 minutes. After washing, the ECs were incubated with the anti‐NF‐κB p65 rabbit mAb (1:100) overnight at 4°C, and then, the ECs were incubated with the Alexa Fluor 594‐conjugated donkey anti‐goat secondary antibody (1:500) for 2 hours. Finally, MPMECs were sealed by ProLong Gold Antifade Mountant with DAPI and were imaged with confocal microscopy. For CD31 staining in the lung sections, after incubation with anti‐CD31 antibody, Alexa Fluor 555‐conjugated goat antimouse secondary antibody was used.

### 2.8 Animal studies (ALI model, lung histopathology, wet/dry ratios, ratios of PaO_2_/FiO_2_ rations and Evans blue extravasation)

Male C57BL/6 mice at 8 weeks old (20 g) were obtained from the Experimental Animal Center of Ruijin Hospital, Shanghai, China. All animal care and procedures were performed in accordance with the National Institute of Health Guide for the Care and Use of Laboratory Animals with the approval (SYXK‐2018‐0027) of the Scientific Investigation Board of Shanghai Jiao Tong University School of Medicine, Shanghai, China. The animal feeding environment was as follows: (25°C, 12 hour/12 hour light/dark, 50% humidity, and free access to sterilized food and water). The mice were first pre‐treated with or without BAY11‐7082 by intraperitoneal injection for 2 hours, and then, the sepsis‐associated ALI was induced by the caecal ligation and puncture (CLP) model. To determine the protective effect of GSS, the mice were pre‐treated with or without GSS by intravenous injection for 2 hours, and then, the sepsis model was established. For histological assessment of sepsis‐induced ALI, the mouse lungs were removed and fixed in 10% paraformaldehyde. The paraformaldehyde‐fixed lungs were embedded in paraffin and cut into 5‐μm sections. H&E staining was performed, and the slides of each group were assessed under high‐power fields. For assessment of sepsis‐induced lung oedema, the lung samples were wiped with filter paper, weighed and dried in an oven at 80°C for 24 hours, and then, the tissues were reweighted to measure lung oedema by dry/wet ratios. To evaluate the sepsis‐induced lung vascular leakage, Evans blue dye (30 mL/kg) was injected into the caudal vein 2 hours before the mice were killed. Evans blue accumulated in the mouse lung was checked as before.^18^ For measurement of oxygenation index changes, the carotid arteries were cannulated, and the arterial blood samples were collected for analysis.^19^

### 2.9 Measurement of ECs permeability by EB‐albumin flux

EC barrier leakage was checked by the flux of Evans blue‐labelled albumin (EB‐albumin) across EC monolayers. MPMECs were grown for 2‐3 days to confluence on gelatin‐coated Transwell insects (0.4 μm pore size) in 24‐well plates and then incubated with LPS for 24 hours. Subsequently, 0.75 mL of albumin (4%) was added to the lower compartment of the well, and 0.5 mL of EB‐albumin (0.67 mg/mL) was added to the upper compartment. The volume of EB‐albumin leaked into the lower compartment of Transwell inserts was quantified by testing the absorbance of medium at 595 nm. The trans‐ECs EB‐albumin flux is expressed as the percentage of total EB‐albumin added into the upper compartment.

### 2.10 Statistical analysis

The data are expressed as the mean ± SEM. Statistical analyses were performed using SPSS statistical software version 16.0 (SPSS, Inc). Student's *t* tests and ANOVAs were used to make statistical comparisons between groups. All experiments were repeated at least three times, and the significance was accepted at *P* < .05.

## 3 RESULTS

### 3.1 Effect of GSS on EC cytotoxicity

**Figure 1:**
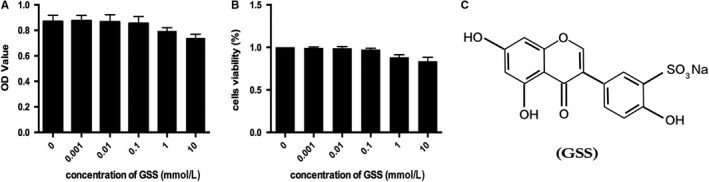
Effect of GSS on EC viability measured by CCK‐8 assay. A, To observe the effect of GSS on the viability of ECs, cells were stimulated with 0, 0.001, 0.01, 0.1, 1 and 10 mmol L^−1^ GSS for 24 h. B, EC viability was measured by CCK‐8 assay. C, The molecular structure of GSS

Based on CCK‐8 cell viability assays, we examined the cytotoxicity of GSS in ECs. As shown in (Figure 1A,B), GSS had no obvious toxic effects on ECs at the concentrations below 0.1 mmol L^−1^. Moreover, although EC cell viability was reduced at concentrations of 1 and 10 mmol L^−1^, the results were not statistically significant compared with the control group. In this study, we further used GSS concentrations at 0.1 mmol L^−1^ to explore its protective effect on LPS‐induced EC apoptosis. Figure 1C showed the molecular structure of GSS.

### 3.2 GSS protects ECs against LPS‐induced apoptosis via BCL‐2

**Figure 2:**
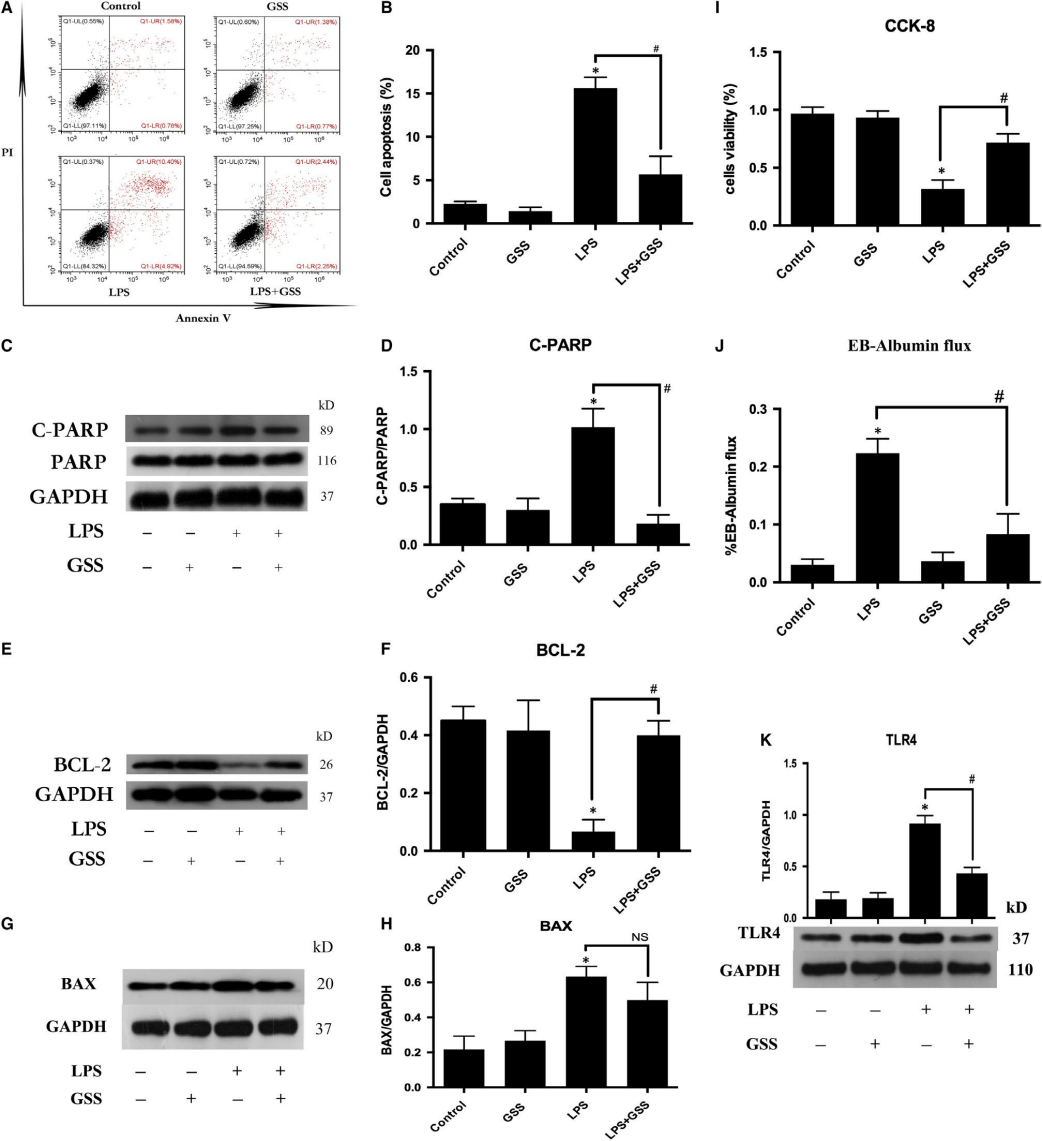
GSS inhibits LPS‐induced EC apoptosis via the TLR4/BCL‐2 signalling. A, ECs were pre‐treated with GSS for 2 h and then exposed to LPS for 24 h, and the apoptotic rate in ECs was detected by Annexin V/PI staining. B, The percentage of apoptotic cells rate is presented as a histogram showing the results obtained by flow cytometry. C, ECs were pre‐treated with GSS for 2 h and then stimulated with LPS for 24 h. The levels of cleaved PARP and total PARP protein were determined by Western blot analysis. D, The Western blot results are presented as a histogram showing the band intensity values. E, After pre‐treatment with GSS for 2 h, ECs were treated with LPS for 24 h, and BCL‐2 expression was detected by Western blot analysis. F, The Western blot results are presented as a histogram showing the band intensity values. G, After pre‐treatment with GSS for 2 h, ECs were treated with LPS for 24 h. The BAX expression was detected by Western blot analysis. H, The Western blot results are presented as a histogram showing the band intensity values. I, ECs were pre‐treated with GSS for 2 h and then exposed to LPS for 24 h. The cell viability rate of ECs was detected by CCK‐8. J, The ECs barrier leakage was checked by the flux of Evans blue‐labelled albumin (EB‐albumin) across ECs monolayers. K, After pre‐treatment with GSS for 2 h, ECs were treated with LPS for 24 h. The TLR4 expression was checked by Western blot analysis. *P < .05 vs negative control. ^#^ *P* < .05 vs the corresponding LPS treatment group

Recent studies have reported that GSS has a protective effect against focal cerebral ischaemia‐induced cytotoxicity in rat cortical neurons by inhibiting cell apoptosis.^17^ To determine whether GSS also protects ECs against LPS‐induced apoptosis, the numbers of apoptotic ECs were quantified by an Annexin V/PI apoptosis kit. We found that LPS‐induced EC apoptosis was significantly blocked by GSS (Figure 2A,B). These data were further confirmed by Western blot analysis of cleaved PARP (Figure 2C,D), which is the substrate of caspase‐3 and is a sensitive marker of apoptosis.^20^ The BCL‐2 family‐mediated intrinsic apoptotic pathway plays a key role in LPS‐induced EC apoptosis.^21^ Genistein inhibits cancer cell apoptosis by regulating the expression of the anti‐apoptotic protein BCL‐2 without altering the expression of the pro‐apoptotic protein BAX.^22^ We further determined the expression changes of BCL‐2 and BAX in LPS‐treated ECs with/without GSS pre‐treatment. Western blot analyses revealed that GSS effectively reversed the LPS‐induced down‐regulation of BCL‐2 (Figure 2E,F); however, the LPS‐induced BAX expression did not be reversed by GSS (Figure 2G,H). Moreover, we also found that GSS inhibited LPS‐induced decrease of cell viability (Figure 2I) and LPS‐induced ECs hyper‐permeability (Figure 2J). GSS also effectively reversed LPS‐induced TLR4 expression (Figure 2K).

### 3.3 GSS attenuates LPS‐induced NF‐κB activation and translocation into the nucleus of ECs

**Figure 3:**
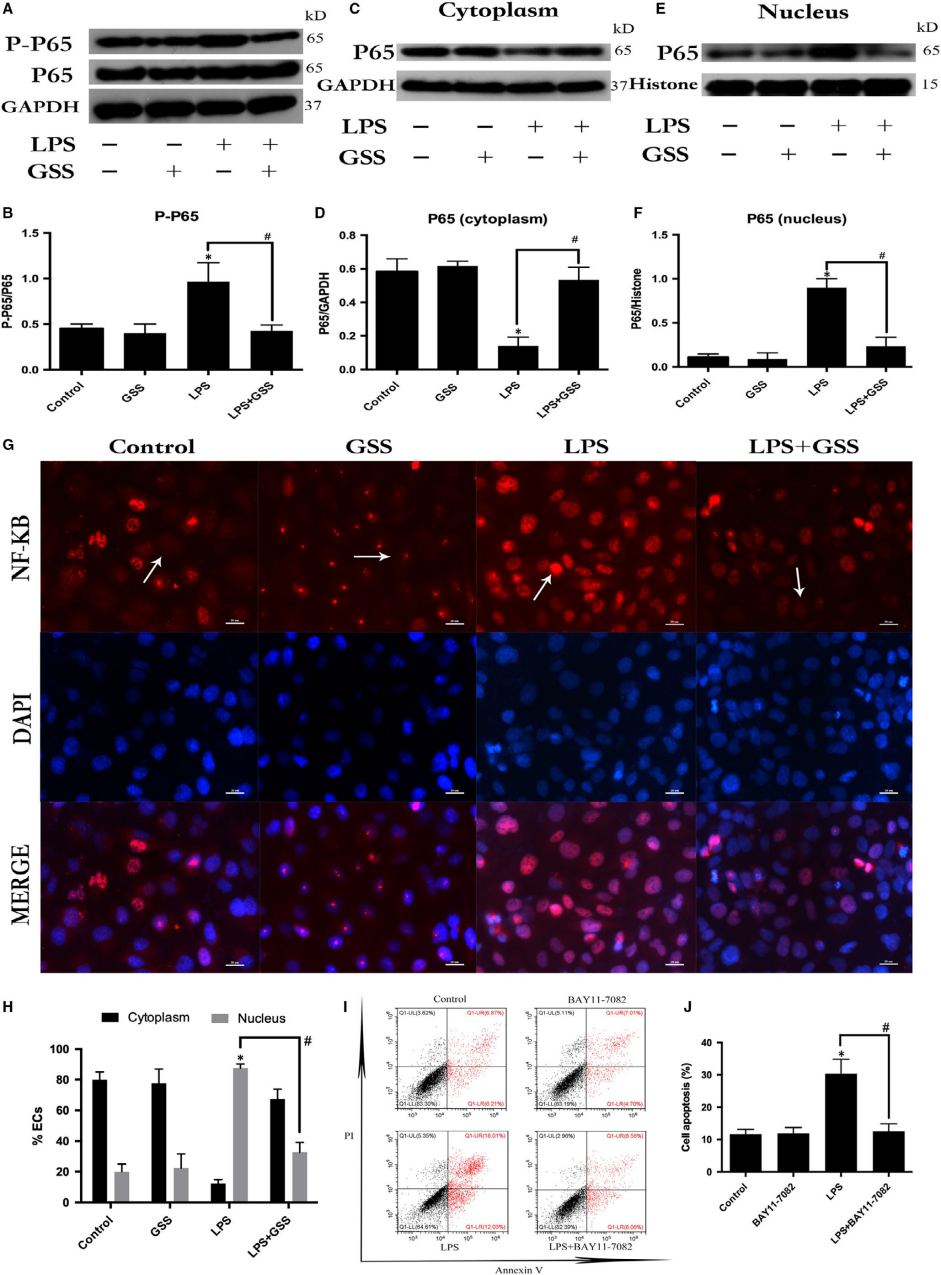
Effects of GSS on the LPS‐induced activation of NF‐κB in ECs. A, Pre‐treatment with GSS for 2 h had a significant inhibitory effect on the LPS‐induced increase in P‐P65 expression compared to the LPS only‐stimulated group (for 1 h). B, The Western blot results are presented as a histogram showing the band intensity values. C, The expression of NF‐κB P65 in the cytoplasm of ECs after incubation with LPS for 1 h was measured by Western blot analysis. D, The Western blot results are presented as a histogram showing the band intensity values. E, NF‐κB P65 expression in the nuclei of ECs after incubation with LPS for 1 h was measured by Western blot assay. F, The Western blot results are presented as a histogram showing the band intensity values. G, The nuclear translocation of NF‐κB was analysed using the confocal microscopy (×200 magnification), which revealed that GSS effectively blocked LPS‐induced NF‐κB P65 translocation into the nucleus of ECs. H, Immunofluorescence analysis of NF‐κB localization. The percentage of ECs showing NF‐κB translocation into nucleus was assessed by counting the number of nuclear ECs. I, ECs were pre‐treated with BAY11‐7082 for 2 h and then exposed to LPS for 24 h, and apoptotic rate of ECs was detected by Annexin V/PI staining. J, The percentage of apoptotic rate of ECs is presented as histogram showing result obtained by flow cytometry. *P < .05 vs negative control. ^#^ *P* < .05 vs the corresponding LPS treatment group

NF‐κB is an important transcription factor and has been implicated in the regulation of EC apoptosis.^23^ To examine whether GSS inhibited LPS‐induced EC apoptosis through NF‐κB signalling, we measured the expression and activation of NF‐κB in ECs. In this study, the ECs were pre‐treated with GSS for 2 hours and then stimulated with LPS for 24 hours. Western blot analysis was conducted to examine the effects of GSS on the LPS‐induced up‐regulation of NF‐κB proteins in ECs. The results showed that GSS exhibited a significant inhibitory effect on the LPS‐induced increase in NF‐κB expression (Figure 3A,B). NF‐κB is an important transcription factor, and the activation of NF‐κB mediates its translocation from the cytoplasm to the nucleus. To examine the effect of GSS on LPS‐induced NF‐κB activation, the nuclear and cytosolic extracts were isolated from ECs. The expression of NF‐κB in the cytosolic and nuclear fractions of LPS‐induced ECs after treatment with/without GSS were determined using Western blot analysis. We found that although LPS increased the nuclear translocation of NF‐κB, pre‐treatment with GSS for 2 hours prior to LPS stimulation significantly decreased the levels of NF‐κB in the nucleus of ECs (Figure 3C‐F). We further used fluorescence confocal microscopy to examine the subcellular localization and fluorescence intensity of NF‐κB. We found that NF‐κB was mainly localized in the nucleus of ECs in the LPS‐stimulated group. However, pre‐incubation with GSS effectively decreased LPS‐induced NF‐κB translocation into the nucleus (Figure 3G,H). Moreover, inhibition the activation of NF‐κB effectively blocked LPS‐induced EC apoptosis (Figure 3J).

### 3.4 GSS does not regulate LPS‐induced GEF‐H1 expression but regulates LPS‐induced Myd88 activation in ECs

**Figure 4:**
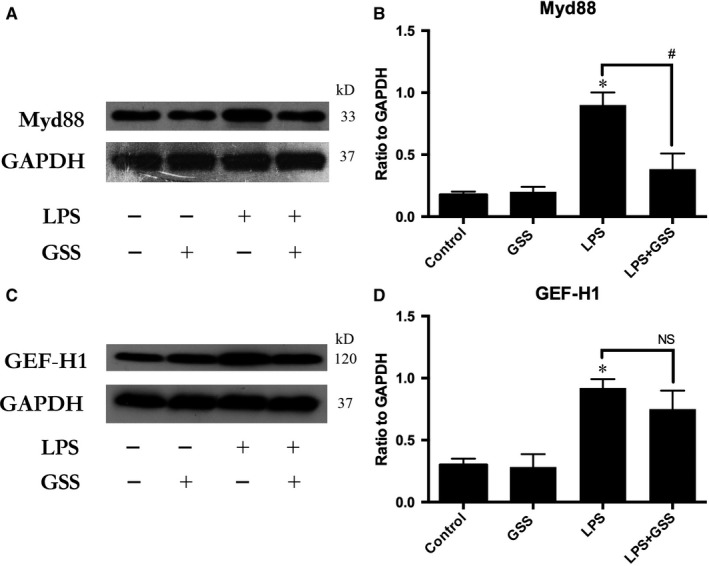
GSS does not inhibit LPS‐induced GEF‐H1 activation but blocks LPS‐induced Myd88 expression. A, ECs were incubated with GSS for 2 h before LPS stimulation for 24 h, and the expression of Myd88 was determined by Western blot. B, The Western blot results are presented as a histogram showing the band intensity values. C, ECs were pre‐treated with GSS for 2 h followed by LPS stimulation for 24 h, and the inhibitory effect on GEF‐H1 was determined by Western blot. D, The Western blot results are presented as a histogram showing the band intensity values. *P < .05 vs negative control. ^#^ *P* < .05 vs the corresponding LPS treatment group

NF‐κB activation is mediated by a wide variety of signals. Our previous studies found that both GEF‐H1 and Myd88 proteins are the upstream of NF‐κB during the process of LPS‐induced EC injury.^24^ To investigate the underlying mechanism of action of GSS on LPS‐induced NF‐κB activation, we incubated ECs with GSS for 2 hours and then added LPS. We performed a Western blot assay to verify whether GSS had an inhibitory effect on GEF‐H1 and Myd88. We found that GSS effectively inhibited LPS‐induced Myd88 expression (Figure 4A,B). In addition, although we found that GSS also blocked the expression of GEF‐H1, the results were not significant (Figure 4C,D). The above data indicated that GSS might protect against LPS‐induced EC apoptosis via Myd88/NF‐κB signalling.

### 3.5 GSS protects against LPS‐induced EC apoptosis by regulating the Myd88/NF‐κB/BCL‐2 signalling pathway

**Figure 5:**
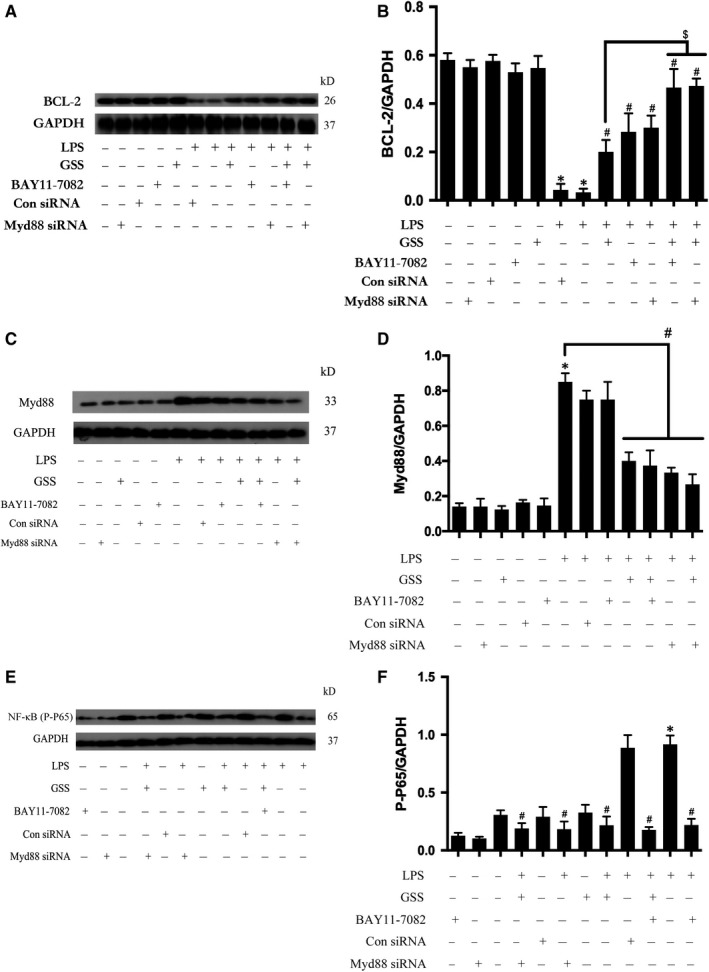
GSS inhibits LPS‐induced EC apoptosis via the Myd88/NF‐κB/BCL‐2 signalling pathway. A, After transfection with Myd88 siRNA for 48 h, ECs were treated with GSS for another 2 h prior to the stimulation with LPS for 24 h, and the expression of BCL‐2 was determined by Western blot. Similarly, ECs were pre‐treated with BAY11‐7082 and GSS for 2 h and then incubated with LPS for 24 h, and BCL‐2 expression was measured by immunoblotting. B, The Western blotting results are presented as a histogram showing the band intensity values. C, After transfection with Myd88 siRNA for 48 h, ECs were treated with GSS for another 2 h prior to the stimulation with LPS for 24 h, and the expression of Myd88 was determined by Western blot. Similarly, ECs were pre‐treated with BAY11‐7082 and GSS for 2 h and then incubated with LPS for 24 h, and the Myd88 expression was checked by immunoblotting. D, The Western blotting results are presented as a histogram showing the band intensity values. E, After transfection with Myd88 siRNA for 48 h, ECs were treated with GSS for another 2 h prior to the stimulation of LPS for 24 h, and the activation of NF‐κB was determined by Western blot. Similarly, ECs were pre‐treated with BAY11‐7082 and GSS for 2 h and then incubated with LPS for 24 h, and the activation of NF‐κB was checked by immunoblotting. F, The Western blotting results are presented as a histogram showing the band intensity values. *P < .05 vs the negative control. ^#^ *P* < .05 vs the corresponding LPS treatment group. ^$^ *P* < .05 vs the corresponding GSS treatment group

To further clarify the protective role of GSS on LPS‐induced EC apoptosis, we pre‐treated ECs with GSS, Myd88 siRNA and BAY11‐7082 (a specific inhibitor of NF‐κB) before stimulation with LPS. As shown in (Figure 5A,B), not only GSS but also inhibition of Myd88 or NF‐κB significantly reversed the LPS‐induced decrease in BCL‐2. Moreover, we also found that co‐pretreatment with GSS and BAY11‐7082 or GSS and Myd88 siRNA increased the reversal effects on the LPS‐induced BCL‐2 decrease. The above data indicate that GSS partially protects ECs against LPS‐induced apoptosis via inactivation of the Myd88/NF‐κB/BCL‐2 signalling pathway. Moreover, we also examined the expression to Myd88 and activity of NF‐κB in different groups in Figure 5F, and we found that LPS regulated the expression of BCL‐2 in ECs via Myd88/NF‐κB signalling pathway.

### 3.6 GSS attenuates sepsis‐induced lung vascular EC apoptosis via TLR4/Myd88/ NF‐κB/BCL‐2 signalling

**Figure 6:**
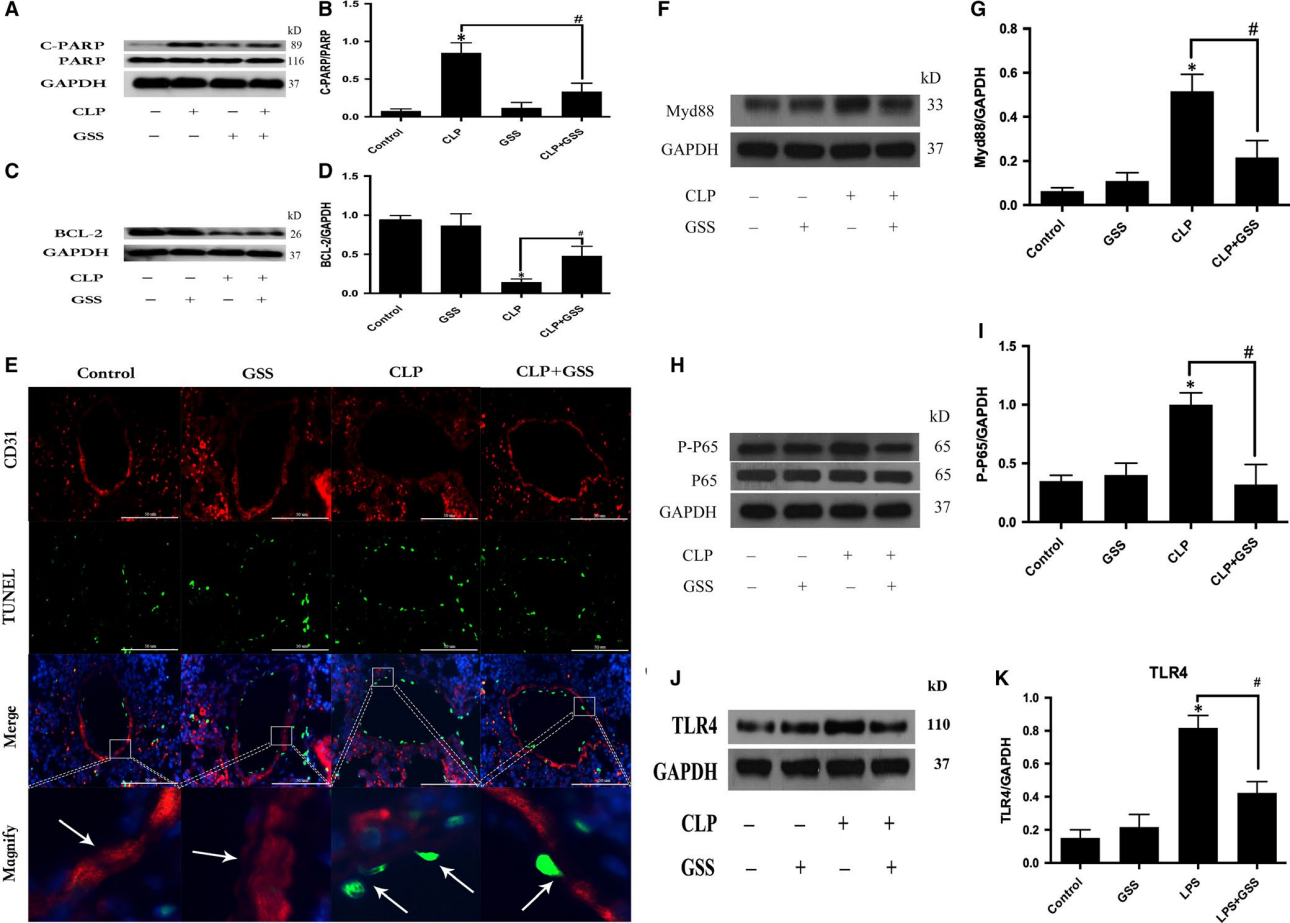
GSS ameliorates sepsis‐induced lung vascular endothelial apoptosis though the BCL‐2 protein. Mouse lungs were harvested 24 h after CLP application. A, The expression of cleaved PARP and total PARP in lung tissue samples was evaluated by Western blot. B, The Western blot results are presented as a histogram showing the band intensity values. C, Expression of BCL‐2 in lung tissue samples was evaluated by Western blot. D, The Western blot results are presented as a histogram showing the band intensity values. E, The lung vascular endothelium and apoptotic cells were stained for CD31 (red) and TUNEL (green), and the results were visualized by immunofluorescence microscopy. The white arrow in the control group and GSS group represents the complete vascular barrier. The arrow in the CLP group represents vascular barrier disruption and endothelial apoptosis. The arrow in the CLP + GSS group shows that vascular barrier disruption is reversed. F, The expression of Myd88 in lung tissue samples was evaluated by Western blot. G, The Western blot results are presented as a histogram showing the band intensity values. H, The expression of P‐P65 and P65 in lung tissue samples were evaluated by Western blot. I, The Western blot results are presented as a histogram showing the band intensity values. J, The expression of TLR4 in lung tissue samples was evaluated by Western blot. K, The Western blot results are presented as a histogram showing the band intensity values. *P < .05 vs negative control. ^#^ *P* < .05 vs the corresponding LPS treatment group

To further determine whether GSS inhibits sepsis‐induced lung vascular EC apoptosis in vivo, we used immunoblot analysis and immunofluorescence microscopy to determine the changes in lung BCL‐2 expression, caspase‐3 activation and lung vascular EC apoptosis. Compared with the control group, the CLP group exhibited obvious pathologic changes, including caspase‐3 activation and a decrease in BCL‐2 expression in the lung. However, these changes were significantly ameliorated by pre‐treatment with GSS (Figure 6A‐D). Importantly, we found that GSS effectively inhibited sepsis‐induced lung vascular EC apoptosis and lung vascular barrier disruption (Figure 6E). Moreover, we also found that GSS effectively inhibited CLP‐induced activation of TLR4/Myd88/ NF‐κB signalling (Figure 6F‐K).

### 3.7 GSS inhibits sepsis‐induced ALI and lung vascular hyper‐permeability

**Figure 7:**
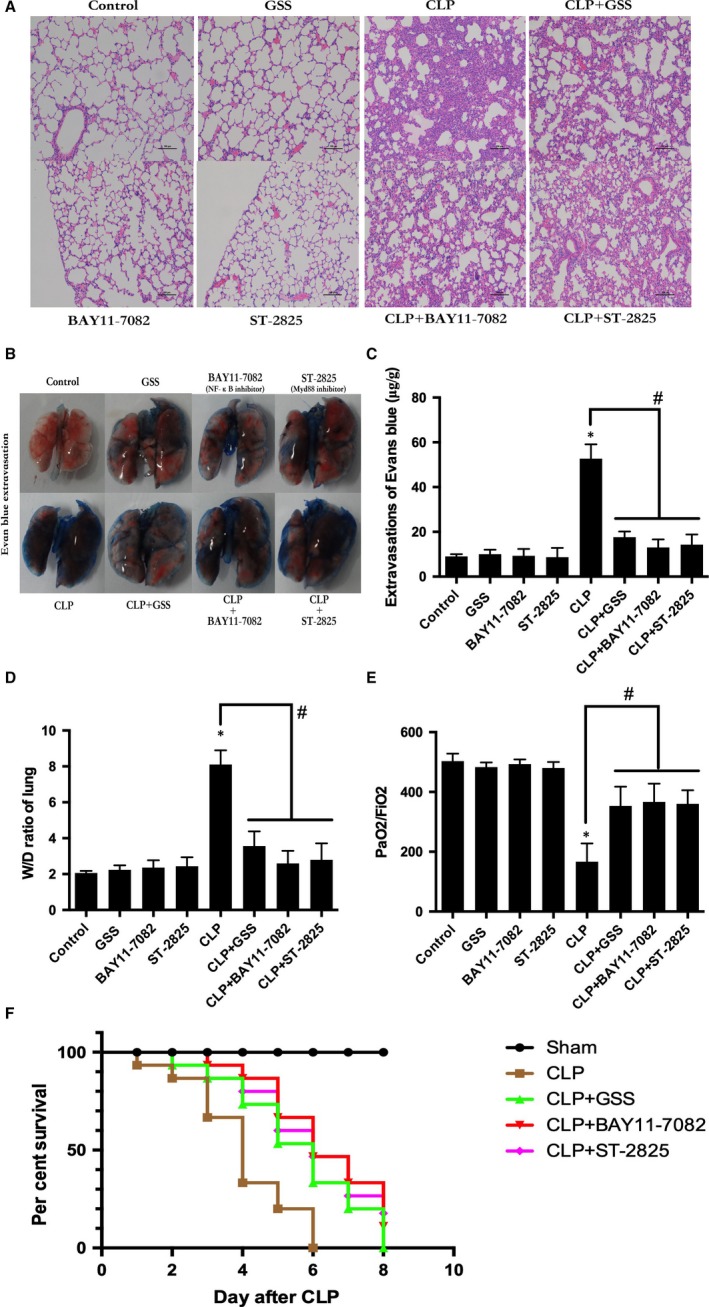
GSS inhibits sepsis‐induced acute lung injury and vascular barrier dysfunction in mice. Mouse lungs were harvested 24 h after CLP application. A, Sepsis in C57BL/6J mice was induced by the CLP procedure after pre‐treatment with GSS or special inhibitor for 2 h, and the histological analysis of lung tissue was analysed by haematoxylin and eosin staining. B, Evans blue dye was injected 2 h before termination of the experiment, and the Evans blue extravasation was photographed in different groups. C, The quantitation of Evans blue extravasation was performed by spectrophotometric analysis of Evans blue extracted from the lung tissue samples. D, Wet/dry ratio of lungs was represented as a histogram according to the collected data. E, The PaO2/FiO2 ratio was detected after CLP in mice with and without GSS or inhibitor. F, The survival rate was detected after CLP in mice with and without GSS or special inhibitor. *P < .05 vs negative control. ^#^ *P* < .05 vs the corresponding LPS treatment group

Lung vascular EC apoptosis could increase lung vascular leakage and induce ALI. To determine whether GSS blocks sepsis‐induced lung vascular barrier disruption and ALI, the effects of GSS on sepsis‐induced lung histological changes were first examined in this study. We found that pre‐treatment with GSS obviously ameliorated sepsis‐induced lung pathologic changes, mainly including abundant inflammatory cell infiltration and interstitial oedema (Figure 7A). Moreover, the effects of GSS on sepsis‐induced lung vascular barrier disruption were detected by the Evans blue dye accumulation (Figure 7B,C). In this study, we also found that GSS reversed the increase in sepsis‐induced wet/dry weight ratios (Figure 7D), the decrease in sepsis‐induced PaO_2_/FiO_2_ ratios (Figure 7E) and the decrease in sepsis‐induced survival rate in mouse lungs (Figure 7F).

## 4 DISCUSSION

ALI is a life‐threatening clinical condition with high mortality. Although many previous studies have revealed the molecular mechanisms of sepsis‐induced ALI,^25^, ^26^ the drugs used to treat ALI have too many side effects to be widely used in the clinic. Natural flavones occur in high concentrations in fruits, which have potent biological activity and have attracted attention. In this study, we demonstrated that one derivative of a natural flavone, GSS, has therapeutic effects on sepsis‐induced ALI via the inhibition of lung vascular EC apoptosis. We further revealed that the underlying anti‐apoptotic mechanisms of GSS were mainly based on inhibition of the LPS‐induced activation of the Myd88/NF‐κB signalling pathway in ECs, which consequently reversed the decrease in BCL‐2 expression and activation of caspase‐3.

Under septic conditions, the lungs are the most affected organs. The LPS released from bacteria into the blood easily injures the lung micro‐vascular ECs,^27^, ^28^ and the lung vascular EC apoptosis complicates sepsis. The apoptotic lung vascular ECs detach from vessel walls in sepsis, resulting in hyper‐permeability of the microvasculature, which promotes the development of ALI.^29^ Therefore, inhibition of lung EC apoptosis is an effective therapeutic strategy for sepsis‐induced ALI.

Flavones are a class of flavonoids, and genistein is a phytoestrogen and a potent natural tyrosine kinase inhibitor.^30^ Previous studies have investigated the role of genistein in brain injury and found that genistein has significant anti‐inflammatory and anti‐oxidative properties.^31^, ^32^, ^33^ However, the poor water solubility of genistein restricted its wide use in the clinic. GSS is a synthetic derivative of genistein, which has more bioavailability than genistein. However, the contribution of GSS to the response to sepsis‐induced apoptosis remains unclear. Interestingly, a recent study reported that GSS could protect cortical neurons from focal cerebral ischaemia‐induced apoptosis of neurons.^17^ To explore the role of GSS on LPS‐induced lung vascular EC apoptosis, we first explored the cytotoxicity of GSS in ECs using CCK‐8 assays. The results demonstrated that although GSS slightly decreased cell viability in a dose‐dependent manner, it had no obvious cytotoxicity to ECs. It is well known that LPS incubation significantly induces EC apoptosis.^34^ In this study, we found that pre‐treatment with GSS effectively reversed LPS‐induced EC apoptosis in vitro. These data provide the first evidence that GSS has significant anti‐apoptotic properties in LPS‐stimulated ECs.

The intrinsic apoptotic pathway is mainly regulated by BCL‐2 family proteins and caspase family proteins that play an important role in LPS‐induced lung endothelial cell apoptosis.^5^ A previous study demonstrated that genistein inversely affects cancer cells apoptosis via regulation of BCL‐2.^22^ However, whether GSS regulated BCL‐2 expression in ECs after stimulation with LPS was unknown. Here, we explored the effects of GSS on BCL‐2 expression and caspase‐3 activation. Analogous to the previous study, our results showed that GSS effectively reversed the LPS‐induced down‐regulation of BCL‐2 and activation of caspase‐3 in ECs. These data indicate that GSS plays a protective role in LPS‐induced lung EC apoptosis via the BCL‐2‐mediated intrinsic apoptotic pathway.

NF‐κB is a critical transcription factor that plays an important role in the regulation of apoptotic genes and is believed to be a promising target for the treatment of apoptosis.^35^ Under normal conditions, NF‐κB exists in an inactivated form and is primarily present in the cytoplasm. When it is activated by phosphorylation, NF‐κB translocates into the nucleus, where active NF‐κB binds to specific DNA sequences and regulates the expression of many downstream genes, such as BCL‐2.^8^ In the present study, we further found that GSS inhibited the activation of NF‐κB and attenuated LPS‐induced NF‐κB translocation from the cytoplasm into the nucleus in ECs. Our data suggested that NF‐κB/BCL‐2 signalling might be involved in the GSS‐induced anti‐apoptotic effects on LPS‐stimulated ECs in vitro. However, the possible upstream proteins of NF‐κB/BCL‐2 signalling involved in the anti‐apoptotic effects of GSS in ECs are unclear. Importantly, our previous studies showed that both Myd88‐dependent signals and Myd88‐independent signals (GEF‐H1‐dependent manner) are the upstream activators of NF‐κB, which plays a critical role in the regulation of LPS‐induced ECs inflammatory injury.^10^, ^11^, ^24^ In addition, Myd88 and GEF‐H1 signalling also correlated with the induction of cellular apoptosis.^13^, ^36^ In light of the indirect correlation between the above signalling pathways and the LPS‐induced EC apoptosis process, we further analysed the role of GSS in the changes in the activities of the Myd88 and GEF‐H1 signalling pathways in LPS‐treated ECs. Here, we found that GSS did not inhibit LPS‐induced GEF‐H1 expression but reversed LPS‐induced overexpression of Myd88 in ECs. Hence, the above data suggest that GSS might protect against LPS‐induced EC apoptosis via the regulation of the Myd88/NF‐κB/BCL‐2 signalling pathway. To further verify whether Myd88/NF‐κB/BCL‐2 signalling is involved in the GSS‐induced anti‐apoptotic effects on LPS‐induced EC apoptosis, we combined the application of GSS, NF‐κB inhibitor and Myd88 siRNA and then studied the changes in downstream BCL‐2 expression. We found that either co‐treatment of GSS with the NF‐κB inhibitor or co‐treatment of GSS with the Myd88 siRNA synergistically reversed the LPS‐induced decrease in BCL‐2. These results suggest that GSS partially restored the LPS‐induced EC apoptosis via the regulation of the Myd88/NF‐κB/BCL‐2 signalling pathway.

To further verify the protective role of GSS on the sepsis‐induced apoptosis of lung vascular ECs in vivo, we used the CLP‐induced murine sepsis model to investigate the therapeutic role of GSS. We found that GSS effectively inhibited the sepsis‐induced BCL‐2 decrease and caspase‐3 activation in lung tissue. Moreover, we subsequently found that the sepsis‐induced apoptosis of murine lung vascular ECs was significantly reduced by GSS. These data suggested that GSS effectively protected the lung vascular endothelium against apoptosis in sepsis. Because lung vascular EC apoptosis plays an important role in the initiation and development of ALI, we further examined the role of GSS in sepsis‐induced ALI. In this study, we found that GSS reversed the sepsis‐induced increase in wet/dry ratios and lung pathological changes, such as pulmonary oedema and neutrophil emigration. Moreover, we also found that GSS effectively blocked the decrease in the oxygen index and survival rate. These results revealed that GSS had an obviously protective role against sepsis‐induced ALI. Both the in vivo and the in vitro data suggest that the GSS has anti‐apoptotic effects on LPS‐induced lung vascular EC injury via TLR4/ Myd88/NF‐κB/BCL‐2 signalling, which may be the main reason why GSS effectively alleviates sepsis‐induced ALI.

## 5 CONCLUSION

**Figure 8:**
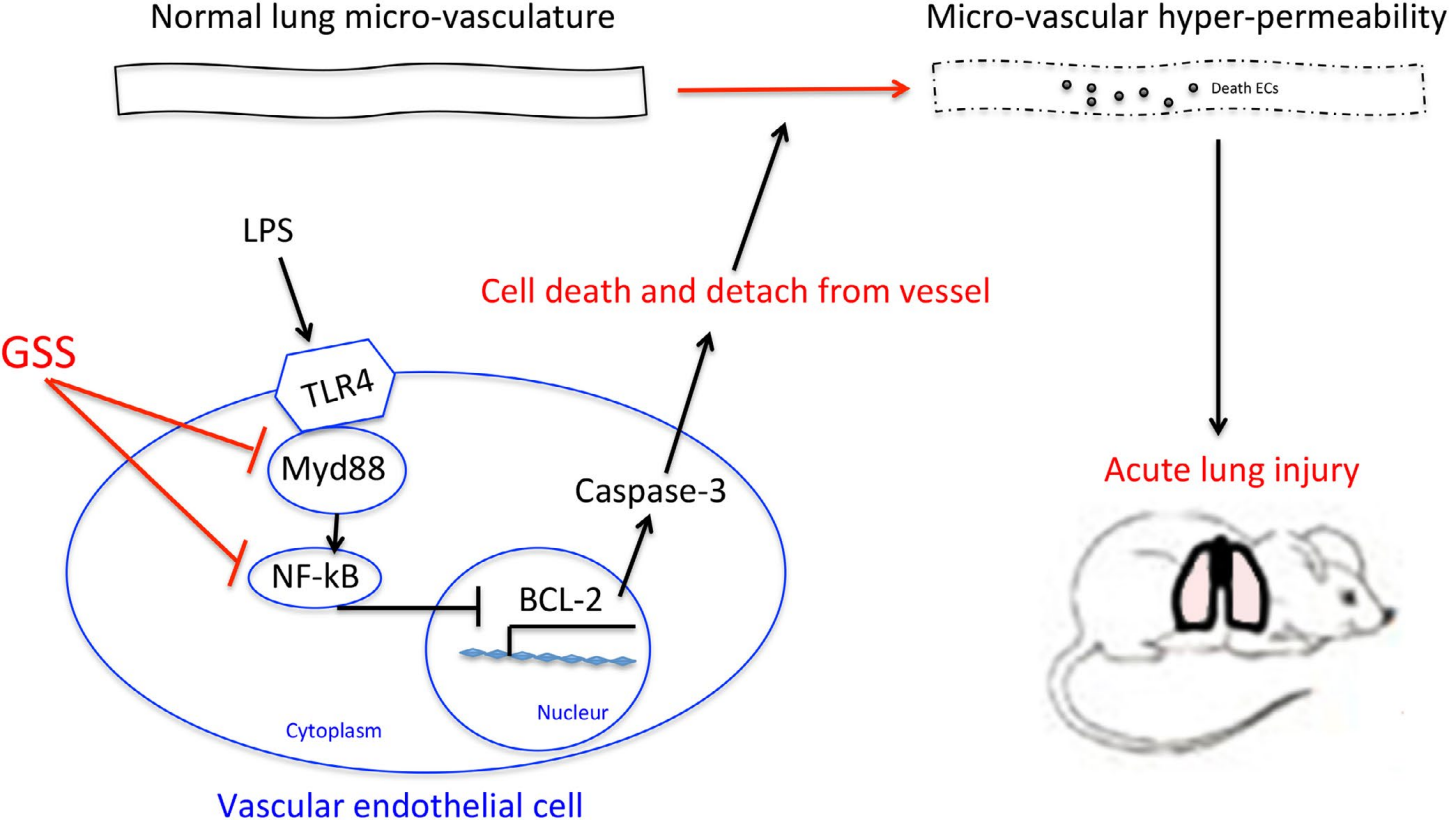
Schematic figure of possible signalling mechanisms of GSS in the inhibition of LPS‐induced apoptosis of lung vascular endothelial cells and subsequent ALI in mice

In this study, we explored the protective role of GSS on lung vascular ECs and ALI in sepsis for the first time. We provide new evidence that the protective mechanism of GSS on LPS‐induced lung EC apoptosis is partially mediated by the Myd88/NF‐κB/BCL‐2 signalling pathway. We also found that GSS significantly attenuated sepsis‐induced ALI by blocking the apoptosis of lung vascular ECs and the subsequent lung vascular barrier disruption. In summary, the present study indicated that GSS is safe and has significant protective effects against lung vascular EC apoptosis and ALI under septic conditions (Figure 8). Taken together, these results suggest that GSS may be a new therapeutic agent for the prevention of sepsis‐induced ALI through inhibition of the apoptosis of lung vascular ECs. Sepsis‐induced ALI began to develop accompanied by the injury of lung epithelial cells and vascular ECs.^37^ In addition to LPS‐induced EC apoptosis, LPS‐induced apoptosis and injury in epithelial cells also play an important role in the process of sepsis‐induced ALI.^38^ Hence, GSS might also block LPS‐induced lung epithelial cell apoptosis, which needs to be studied further. Moreover, LPS‐induced activation of macrophages also plays a very important role in sepsis‐induced organ injury,^39^ whether GSS protects against LPS‐induced activation in macrophages in sepsis condition also needs to be studied further.

## CONFLICT OF INTEREST

The authors confirm that there are no conflicts of interest.

## AUTHORS’ CONTRIBUTIONS

Lei Yi and Mengling Chang drafted the manuscript. Lei Yi, Feng Guo and Jingning Huan conserved the proposal, revised the manuscript and provided funding support. Quanming Zhao, Zengding Zhou and Xiaoqin Huang made substantial contributions to acquisition of data and editing the manuscript.

## Data Availability

The data that support the findings of this study are openly available in [repository name, eg ‘figshare’] at http://doi.org/%5Bdoi], reference number [reference number].
